# Enzootic situation and molecular epidemiology of *Brucella* in livestock from 2011 to 2015 in Qingyang, China

**DOI:** 10.1038/s41426-018-0060-y

**Published:** 2018-04-04

**Authors:** Xiaoan Cao, Shien Li, Zhaocai Li, Zhijun Liu, Jie Ma, Zhongzi Lou, Jizhang Zhou, Yongsheng Liu, Zhizhong Jing, Baoquan Fu

**Affiliations:** 1 State Key Laboratory of Veterinary Etiological Biology, Lanzhou Veterinary Research Institute, Chinese Academy of Agricultural Sciences, Lanzhou, 730046 P.R. China; 2 Qingyang Center for Animal Disease Prevention and Control, Qingyang, 745000 P.R. China; 3 Huanxian Center for Animal Disease Prevention and Control, Huanxian, 745700 P.R. China; 4 Qingcheng Center for Animal Disease Prevention and Control, Qingcheng, 745100 P.R. China

## Abstract

A large-scale survey was conducted in domestic animal populations from 2011 to 2015 in Qingyang, China. A total of 448,398 animals from different districts of Qingyang were tested for the presence of *Brucella*-specific antibodies using the Rose Bengal Plate Test (RBPT) and the Standard Agglutination Test (SAT). From 2011 to 2015, the yearly average positive rates were between 0.04 and 4.75% in the eight counties tested. In addition, the prevalence rates were between 0 and 9.96% in these eight counties. Sheep was the dominant host of *Brucella* in Qingyang, and the prevalence rate in sheep (2.74%) was higher than those in the other animals tested. Identification of 10 *Brucella* isolates from sheep confirmed that the epidemic strains were *B. melitensis* biovar 3 (*n* = 9) and *B. melitensis* biovar 1 (*n* = 1). MLVA-11 (multiple-locus variable-number tandem repeat analysis) analysis of the 10 isolates showed three genotypes: genotype 116 (*n* = 8), genotype 115 (*n* = 1) and genotype 136 (*n* = 1). Furthermore, analysis of the whole-genome sequences of the representative *B. melitensis* strain QY1 indicated that this isolate was closely related to isolates from China and India. The results of serum epidemiology confirmed that the region of northern Qingyang was a critical *Brucella* epidemic area and that the disease showed a rising trend, especially from 2013 to 2015. An analysis of the isolate genotypes suggested that sheep brucellosis mainly resulted from conventional *B. melitensis* (East Mediterranean group), although the external strain (American group) also occurred in Qingyang.

## Introduction

Brucellosis, caused by *Brucella spp*., is a zoonotic bacterial disease that has serious implications for human and animal health^1^. It is a highly contagious disease that affects livestock such as cattle, sheep and pigs. The symptoms include abortion, infertility, decreased production and lameness in animals and undulating fever with arthralgia in humans^2^. Recently, this disease has re-emerged in some regions of China^3–6^. From an epidemiological perspective, the major cases in animals have been sheep infected by *B. melitensis*. Human brucellosis has re-occurred in the past several years^7,8^. In Qingyang, four cases were detected from 2006 to 2008, while from 2009 to 2012, the number of positively detected *Brucella* cases was between 25 and 57. In 2013 and 2014, however, the cases of human brucellosis numbered 161 and 457, respectively^9^. Among animal cases, from 2009 to 2013, the seroprevalence was <1%, although brucellosis was already occurring in domestic animals^10^.

Over the past decade, the number of breeding stock of both cattle and swine were approximately 400,000 in Qingyang. However, sheep farming was developed vigorously with main farmers feed. From 2007 to 2013, more than two million sheep were bred, and the number of sheep breeding stock was over 4 million in both 2014 and 2015 in Qingyang. From the beginning of this century to 2016, although animal brucellosis occurred in an increasing number of cases, the local control strategy was to cull the infected animals that presented as serologically *Brucella* positive. After 2016, vaccination was required in the area because of changes in government control policy, which were based on an assessment of the development of brucellosis in animals. Therefore, detailed knowledge of the epidemiological situation has become very important for the vaccination program and for assessment of effective prevention. This study was aimed at understanding the enzootic situation of *Brucella* in livestock and the characteristics of epidemics. Comprehensive *Brucella* seroepidemiological surveys were performed in livestock epidemics of brucellosis from 2011 to 2015 in Qingyang, China. To further illuminate the molecular features of epidemic *Brucella*, bacterial isolation and whole-genome sequencing were also performed.

## Results

### Domestic brucellosis in Qingyang from 2011 to 2015

**Fig. 1:**
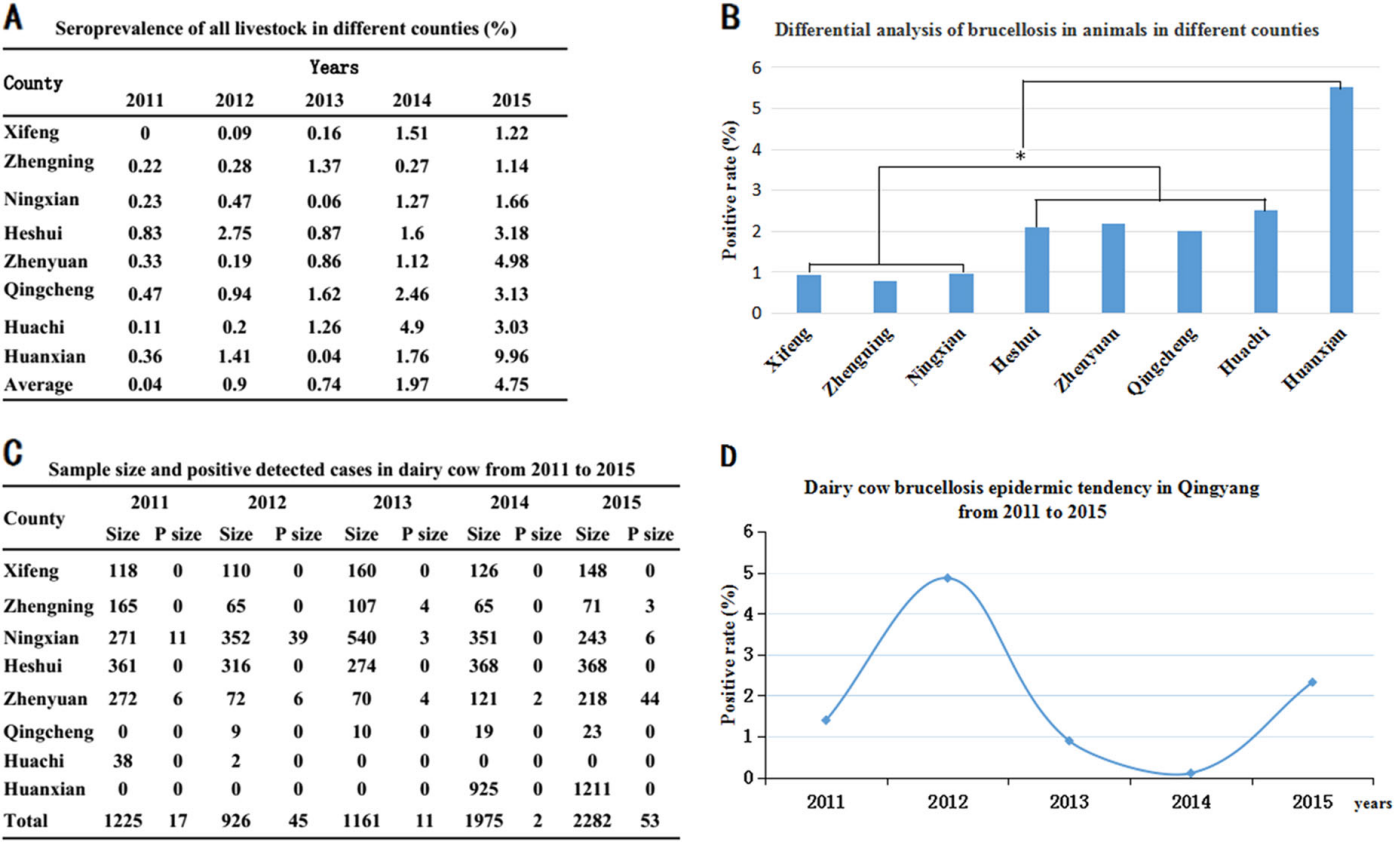
The seroprevalence of brucellosis in livestock in Qingyang from 2011 to 2015. **a** The positive rates of the different counties in each year. **b** Differential analysis of brucellosis in different counties from 2011 to 2015. Bar, the average seroprevalence from 2011 to 2015; *Significant difference at *P* < 0.05. **c** Sample sizes and positively detected cases in the dairy cow brucellosis epidemic in Qingyang from 2011 to 2015. **d** Dairy cow brucellosis epidemic tendency in Qingyang from 2011 to 2015

Of a total of 448,398 serum samples from different animals, 11,654 (2.6%) were anti-*Brucella* positive. The yearly positive rates in swine, yellow cattle (native breed), dairy cows (Holstein), sheep and dogs were 0%, 0.04%, 1.66%, 2.74% and 0.14%, respectively. Sheep and dairy cows with *Brucella* had higher infection trends, although the positive rates in dairy cows dipped to a downward trend between 2013 and 2014. Compared with that in sheep and dairy cows, brucellosis in yellow cattle and dogs maintained sporadic onset, and no cases were found in swine (Table 1). From 2011 to 2015, the average positive rates in each year were from 0.04 to 4.75% in eight counties. In addition, the yearly prevalence rates were among 0 to 9.96% in these eight counties, and the disease emerged with an obvious rising trend, especially in the years 2013 and 2015. However, brucellosis in animals showed regional differences (Figs. 1a, b). Overall, domestic brucellosis in Qingyang predominantly infected sheep. Huanxian, located in the north Qingyang district, was a serious animal *Brucella* epidemic area. In the years 2013 and 2014, 6 (0.04%) of the 13,793 total yellow cattle were brucellosis positive, and 0.14% (1/698) seroprevalence was detected in dogs (Table 1).

**Table 1:** Sample size and positive number of different animals from 2011 to 2015 years

Years	Swine		Yellow cattle		Dairy cow		Sheep		Dog	
	Size	P size	Size	P size	Size	P size	Size	P size	Size	P size
2011	390	0	1356	0	1225	17	29,141	97	0	0
2012	238	0	881	0	926	45	59,644	509	0	0
2013	1819	0	10,249	2	1306	11	54,872	501	556	1
2014	1440	0	3544	4	1975	2	104,127	2185	140	0
2015	0	0	53	0	2282	53	171,822	8226	2	0
Total	3887	0	16,083	6	7714	128	419,606	11,518	698	1

*Size* sample number collection, *P size* positive number

### Dairy cow brucellosis in Qingyang

Serological examination revealed that dairy cow brucellosis mainly emerged in the southern region of Qingyang, including Ningxian, Zhenyuan and Zhengning counties, whereas no dairy cow *Brucella* cases were found in the northern region of Qingyang. The yearly prevalence rates of *Brucella*-infected dairy cows was from 0.89 to 4.86% from 2011 to 2015 without incidence trends, and the highest positive detection rate was 20.12% in Zhenyuan in 2015 (Figs. 1c, d).

### Sheep brucellosis in Qingyang

**Fig. 2:**
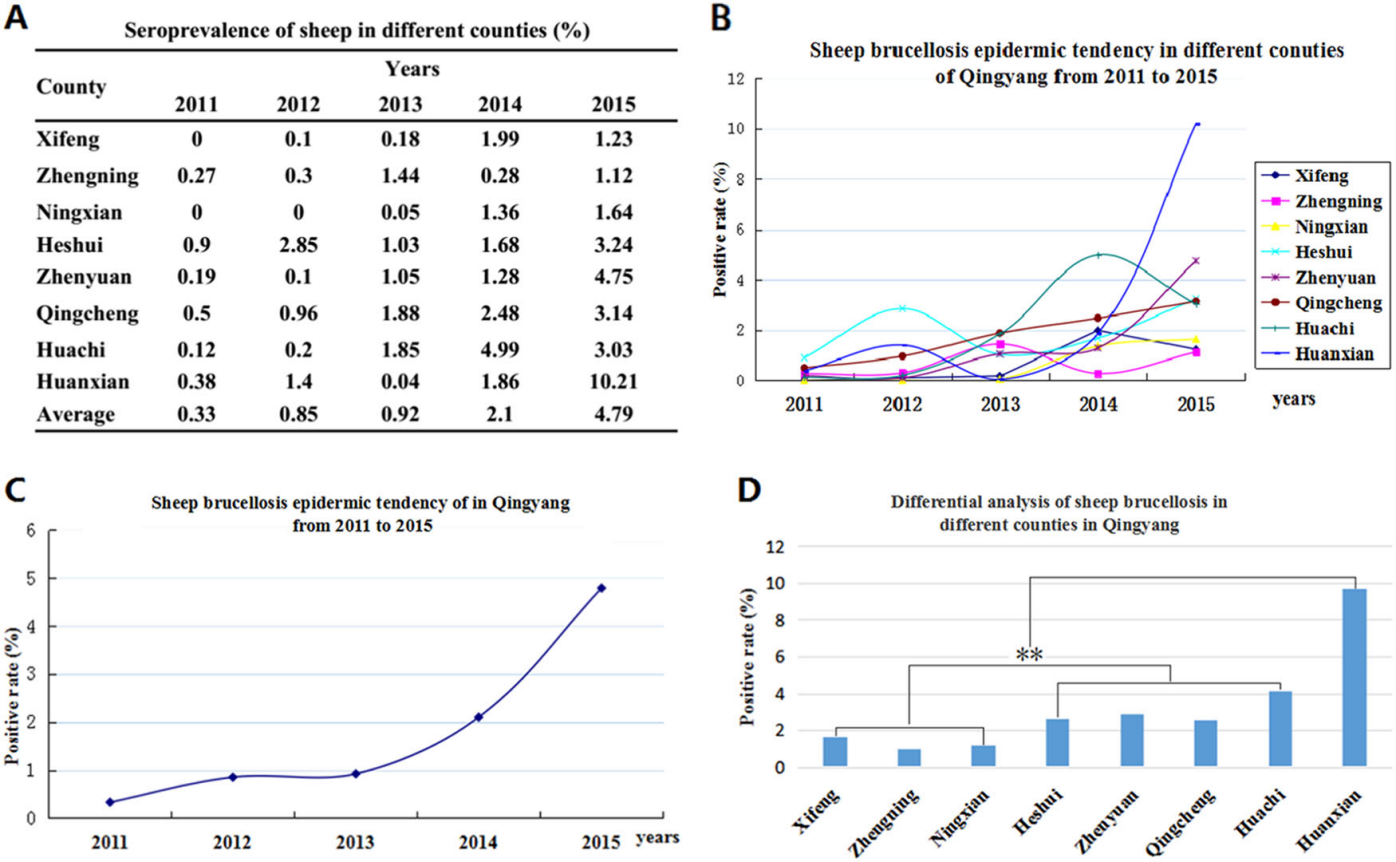
The seroprevalence of brucellosis in sheep from 2011 to 2015. **a** The positive rates of the different counties in each year from 2011 to 2015. **b** Sheep brucellosis epidemic tendency in Qingyang from 2011 to 2015. **c** Sheep brucellosis epidemic tendencies in different counties in Qingyang from 2011 to 2015. **d** Differential analysis of the seroprevalence in different counties in Qingyang. Bar, the average seroprevalence from 2011 to 2015; **Very significant difference at *P* < 0.01

**Fig. 3:**
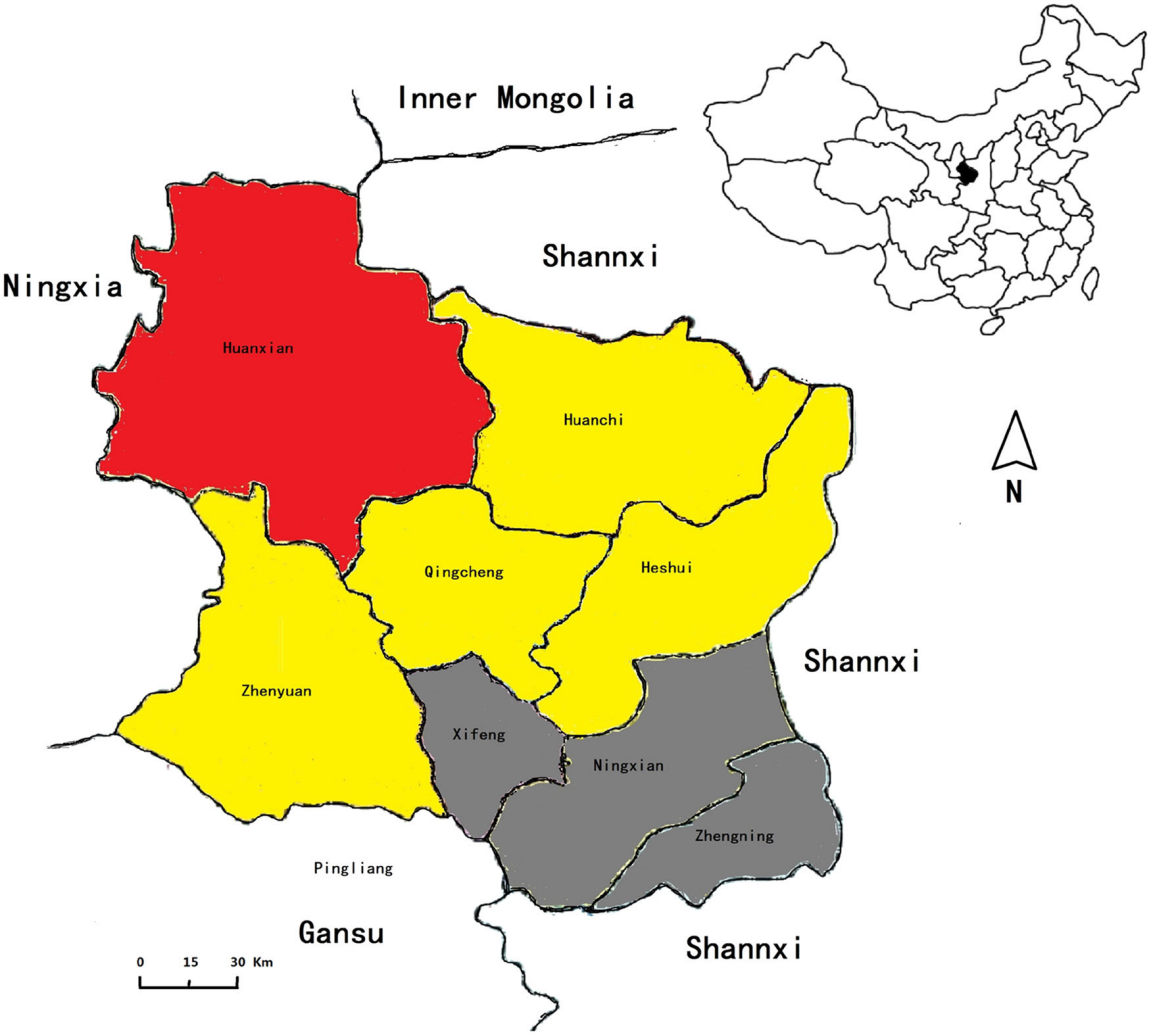
Geographic distribution of the seroprevalence of brucellosis in livestock in Qingyang, China. Red area: seroprevalence of 2.71%; yellow area: seroprevalence between 1.50 and 1.90%; gray area: seroprevalence <1%

To clarify the situation of *Brucella* in sheep in Qingyang district, an analysis of the epidemic in eight counties showed that the *Brucella* prevalence in each year ranged from 0 to 10.21% (Fig. 2), and the average was found to be 0.33 to 4.79% from 2011 to 2015 (Figs. 2a, b). Furthermore, the prevalence of *Brucella* showed an obvious rising trend, and the disease displayed a significant regional difference (Figs. 2c, d). According to the geographical locations of the eight counties, *Brucella* in sheep showed an increasing gradient from south to north. Three southern counties, Zhengning, Ningxian and Xifeng, had lower positive rates (<2%) in sheep, but the positive rate in northern Huanxian was the highest (9.7%). In the other counties, including Zhengyuan, Qingcheng, Huachi and Heshui, the positive rates of brucellosis were from 2.65 to 4.17% (Fig. 3). Although the average seroprevalence in Huanxian was significantly higher than those in other counties, no regional difference (*P* > 0.05) was displayed because of the outbreak in 2015.

### Isolation and identification of *Brucella* strains in sheep

**Fig. 4:**
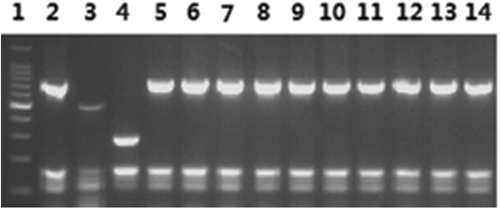
AMOS-PCR of isolates in Qingyang. Lane 1: 100-bp DNA ladder; 2: *B. melitensis* bv. 1 (16 M reference strain); 3: *B. abortus* 544; 4: *B. suis* bv. 1 (S2 vaccine); 5–14: the 10 *B. melitensis* isolates

From 55 spleens of aborted samples, a total of 10 isolates were obtained. Specifically, five and two isolates were isolated from Huanxian and Zhenyuan, and one isolate was isolated from each of Huachi, Heshui and Zhengning. Biochemical testing and AMOS-PCR showed that nine biovars were identified, of which one was *B. melitensis* biovar 1 and nine were *B. melitensis* biovar 3 (*n* = 1; Fig. 4).

### Molecular epidemiological tracking of isolates from sheep

**Fig. 5:**
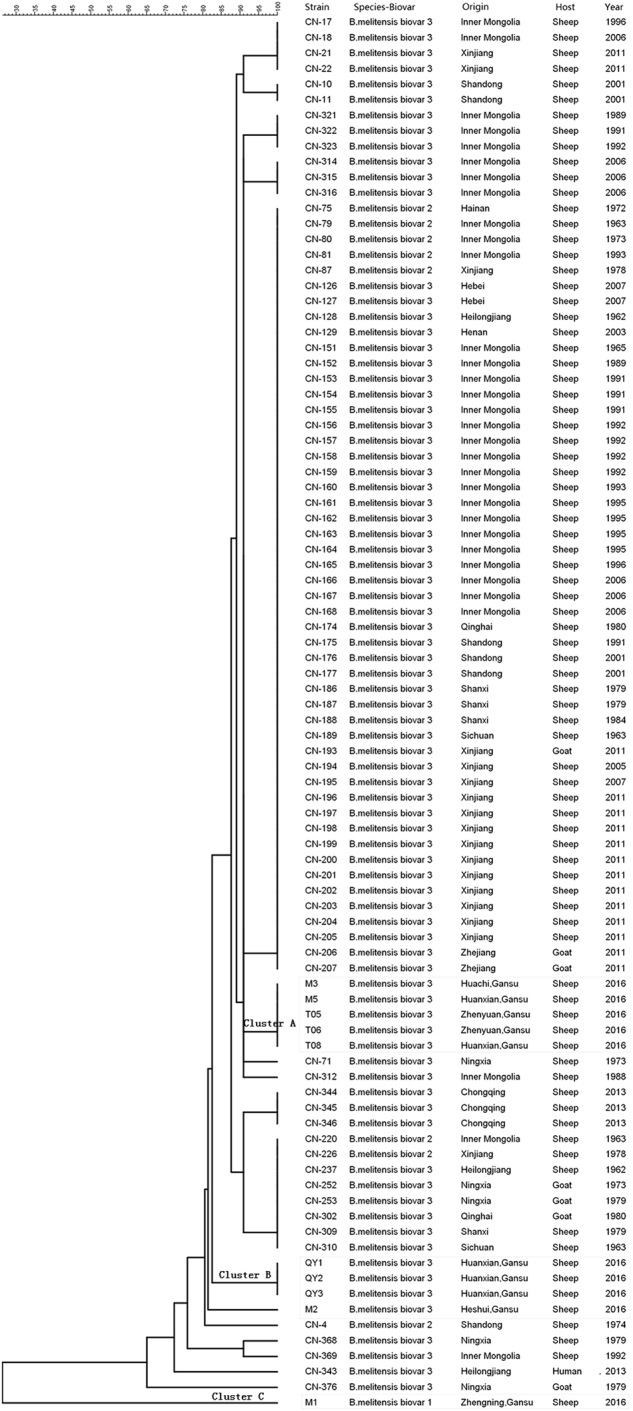
Dendrogram based on the MLVA-11 genotyping assay showing the relationships among the *Brucella* isolates. Strain: isolate number; Origin: sample collection area; Host: host from which the strain was isolated; Year: time of isolation

Analysis of MLVA-16 (multiple-locus variable-number tandem repeat analysis) loci revealed diverse genotypes among the 10 isolates in Qingyang. *B. melitensis* biovar 3 was the predominant biovar in the examined areas. According to the panel 1 markers, all isolates were clustered into four known genotypes: 45 (1-5-3-12-2-2-3-2; *n* = 1), 63 (1-5-13-2-3-3-2; *n* = 3), 42 (1-5-3-13-2-2-3-2; *n* = 5) and 47 (3-4-2-13-4-2-3-3; *n* = 1). The MLVA-11 assay involved 11 loci including Bruce06, Bruce08, Bruce11, Bruce12, Bruce18, Bruce19, Bruce21, Bruce42, Bruce43, Bruce45 and Bruce55. The results of the MLVA-11 analysis showed that the 10 isolates included three genotypes: genotype 116 (*n* = 8), genotype 115 (*n* = 1) and genotype 136 (*n* = 1). To track the source of the infection strains and their evolutionary relationships, MLVA-11 was used to analyze and compare the isolates in Qingyang with *B. melitensis* strains in other provinces, including Shandong, Inner Mongolia, Xinjiang, Ningxia, Hebei, Heilongjiang, Henan, Qinghai, Chongqing, Shanxi, Sichuan and Zhejiang provinces^8^. Comparing these 10 isolates with different *B. melitensis* strains from sheep based on MLVA-11, it was shown that the isolates located in three relatively independent clusters (clusters A, B and C) represented different *B. melitensis* strains from another region (Fig. 5 and Table S1).

### Phylogenetic and evolutionary relationships of *B. melitensis* QY1

**Fig. 6:**
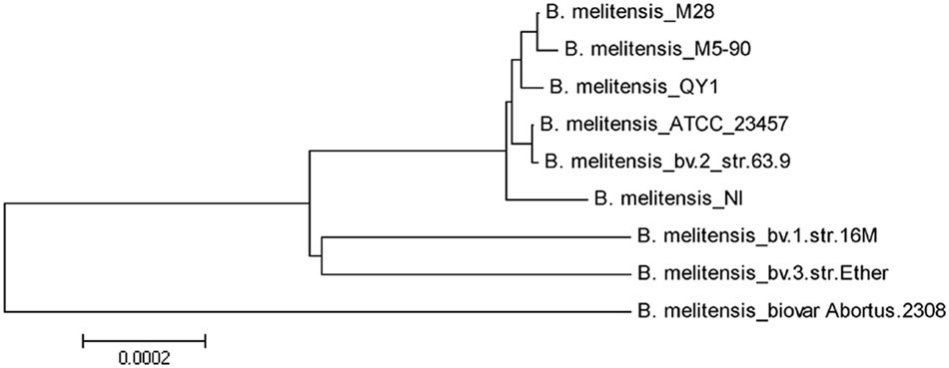
Phylogenetic tree based on the whole genomes of nine selected *B. melitensis* strains. The evolutionary history was inferred using the neighbor-joining method. The optimal tree (with branch length sum = 0.00316668) is shown. The tree is drawn to scale, with branch lengths in the same units as those of the evolutionary distances used to infer the phylogenetic tree

Analysis of the whole-genome sequences showed that *B. melitensis* QY1 has two circular chromosomes with 2,125,648 bp in chromosome I and 1,185,604 bp in chromosome II. The results of a phylogenetic tree based on the whole genome demonstrated that *B. melitensis* QY1 is close to *B. melitensis* M28, *B. melitensis* NI and *B. melitensis* M5-90, which originated in China, as well as *B. melitensis* bv. 3 str. Ether, a strain that was isolated from India (Fig. 6).

## Discussion

Qingyang is located at the intersection of Gansu, Shaanxi and Ningxia provinces and is near Inner Mongolia. The latter two provinces have very large livestock populations with high prevalences of *Brucella*^3,11^. In the present study, we used serological methods to investigate animal brucellosis from 2011 to 2015 in Qingyang, China. The results of seroprevalence assays confirmed that *Brucella spp*. was mainly epidemic in sheep and dairy cows, whereas other animals had a low incidence. The results also showed that the disease has attained an obvious rising trend. Similarly, the number of human cases of brucellosis has also increased^9,12,13^. These results imply that the prevalence of brucellosis in animals, especially in sheep, has caused an increase in the human disease. In contrast, although *Brucella* infected a large number of sheep and dairy cows, yellow cattle presented very few cases.

From 2011 to 2015, brucellosis in dairy cows emerged without incidence trends in Qingyang. There are two possible explanations for this phenomenon. The breeding stock of dairy cows was <3000, so the disease was easy to control. In addition, the local control strategy was to cull infected animals, which largely prevented the expansion of *Brucella* in the short term. From an epidemiological perspective, sheep brucellosis emerged broadly in Qingyang, and dairy cow brucellosis occurred as outbreaks in some dairy farms. It was implied that brucellosis in dairy cows came from infected sheep.

Currently, the number of sheep breeding stock is over 4 million in Qingyang, and almost half of the sheep were raised in Huanxian. From 2011 to 2014, the seroprevalence of brucellosis in Huanxian was <2%, but an outbreak of the disease emerged in the year 2015. The most important factors may be convenient transportation and the prevalence of *Brucella* in most regions of China, as well as monitoring of animals with *Brucella*. Meanwhile, in the past few years, the prevalence of brucellosis in animals and humans has increased each year in Ningxia province, which is adjacent to northern Huanxian^11^. This result may be explained by the fact that frequent livestock exchange occurs between these areas. The outbreak of brucellosis in 2015 further reflected a lack of control in the movement of infected animals between the regions and the supervision of the market. This hypothesis suggests that inspection measures to ensure the quarantine of livestock exported from endemic regions should be strengthened^7^.

To clarify the molecular features of sheep *Brucella* in Qingyang, 10 isolates were revived from Huanxian, Zhenyuan, Huachi, Heshui and Zhengning. Genotyping by MLVA-11 showed that the isolates were divided into three relatively independent clusters (cluster A, B and C; Fig. 5). Cluster A, containing five isolates, was very close to major strains that were responsible for the dominant form of sheep brucellosis in China. However, the isolates in Cluster B were closely related to a few strains isolated from Ningxia, Inner Mongolia, Heilongjiang and Shandong^8^. An isolate in Cluster C belongs to *B. melitensis* biovar 1, which was much closer to *Brucella* strains isolated from Hainan, China, in 2013^8^. The results of MLVA-11 confirmed that major isolates were genotype 116 (*n* = 8), a common genotype derived from *Brucella* strains in the East Mediterranean. Moreover, this genotype was also responsible for the vast majority of *Brucella* infections in China. However, an isolate (genotype 136) from an American lineage was identified and observed in this study. Based on the analysis of these results, it is suggested that sheep brucellosis in Qingyang occurred not only due to a primary epidemic focus but also because of invasion by external bacterial strains with the introduction of sheep.

*B. melitensis* QY1, as a representative strain, was sequenced and analyzed. The whole genome was 3,311,252 bp in size. This study compared *B. melitensis* QY1 with *B. melitensis* M28*, B. melitensis* M5-90, *B. melitensis* NI, *B. melitensis* bv. 1 str. *16M, B. melitensis* bv. 3 str. Ether, *B. melitensis biovar abortus* 2308 and *B. melitensis* ATCC 23457. The results of comparative genomics confirmed that *B. melitensis* QY1 was a dominant pathogenic strain in sheep and had a high identity to *Brucella* strains from China.

Sheep brucellosis re-emerged in Qingyang with rising trends. Therefore, a wide range of vaccination strategies were adopted starting in 2016. China has applied a vaccination program using live attenuated strains S2 (*B. suis* biovar 1 S2) and M5 (*B. melitensis* M5-90)^14–16^, but the problem is to distinguish positive sera due to vaccination from positive sera due to field strain infection. Modified live attenuated vaccines have been developed using differential diagnosis, but so far there are no commercial applications. Studies of molecular epidemiology and strain characteristics can provide support for vaccine selection and development.

## Materials and methods

### Ethics statement

This study involves the investigation and isolation of *Brucella spp*. using serology and modern typing methods. All animal experiments in the study were approved by the Animal Ethics Committee of Lanzhou Veterinary Research Institute, Chinese Academy of Agricultural Sciences, and were conducted strictly according to the guidelines.

### Collection of samples and testing

Serum samples were collected from livestock in Qingyang district, China, from March through July in 2011 to 2015. Samples of aborted sheep fetuses were collected from April through July in both 2015 and 2016. From 2011 to 2015, a total of 448,398 serum samples, including swine (4237), yellow cattle (16,433), dairy cow (7569), sheep (419,461) and dog (698), were collected from Qingyang district, Gansu province, China (Table 1). These samples were collected from eight counties, including Xifeng (32,478), Zhengning (30,904), Ningxian (56,228), Heshui (62,906), Zhenyuan (44,576), Qingcheng (48,212), Huachi (75,396) and Huanxian (98,213) (Fig. 1, Table 1 and Table S2). The sample collection rates in sheep were from 2 to 5% (proportion of total population), the dairy cows were fully sampled, and sampling rates in the other livestock were higher than 20%. All sample collection was performed to cover the full area of Qingyang district, which was sampled randomly. All serum samples were transported to the laboratory, where they were stored at −20 °C until processing and detection by Rose Bengal Plate Test (RBPT), and the positive serum samples were reconfirmed by a Standard Agglutination Test (SAT)^17^ (all the reagents were purchased from China Institute of Veterinary Drug Control).

### Isolation of *Brucella*

To study the molecular epidemiological characters, 55 spleens of aborted sheep were collected at random from eight counties and subjected to a brucellosis purification progress. Spleens were crushed and cultured on *Brucella* serum dextrose agar composed of *Brucella* medium base (supplemented with *Brucella* selective antibiotic, OXOID, England), and 5–10% heat-inactivated horse serum. Plates were incubated with and without 5–10% carbon dioxide at 37 °C after inoculation with sample materials. The plates were examined after 3–15 days for bacterial growth. A single clone was chosen for identification.

### Identification of isolates

The obtained single bacterial clones were identified by biochemical testing according to the standard strain identification method^17–19^. The carbon dioxide (CO_2_) requirement was tested on *Brucella* serum dextrose agar with and without CO_2_ during the first isolation. Agglutination by A, M and R monospecific antisera were detected by mixing the antisera with the isolate after dilution of the colony (all reagents were purchased from The Chinese Center for Disease Control and Prevention). Total genomic DNA was extracted using a DNeasy Blood & Tissue Kit (Qiagen, Germany) according to the manufacturer’s instructions. The DNA extracted from all isolates was stored at −20 °C. AMOS-PCR was performed as previously described^20,21^.

### Molecular epidemiological tracking of isolates by MLVA-16

MLVA was performed as described previously^22–24^. Sixteen primer pairs were included in panel 1 (bruce06, bruce08, bruce11, bruce12, bruce42, bruce43, bruce45 and bruce55), panel 2A (bruce18, bruce19 and bruce21), and panel 2B (bruce04, bruce07, bruce09, bruce16 and bruce30). Polymerase chain reaction (PCR) amplification was performed in a 50 μL reaction system, containing 1 μL DNA template, 1 μL of each primer, 25 μL of 2 × Premix Taq, and 22 μL of ddH_2_O. The reaction conditions were as follows: initial denaturation at 94 °C for 5 min; followed by 40 cycles of 94 °C for 30 s, 60 °C for 30 s and 72 °C for 1 min. At last, the samples were incubated for an additional 10 min at 72 °C and were then stored at 4 °C. Five microliters of each PCR product from panel 1 was loaded onto a 2% agarose gel with 0.5% ethidium bromide, whereas those from panels 2A and 2B were loaded into 3% agarose gels also containing 0.5% ethidium bromide. The bands were visualized under UV light and photographed. The PCR products for the 16 loci were denatured and resolved by capillary electrophoresis on an ABI Prism 3130 automated fluorescent capillary DNA sequencer (Applied Biosystems). Fragments were sized following comparison with a ROX (carboxy-X-rhodamine)-labeled molecular ladder (MapMarker 1000; BioVentures Inc., Murfreesboro, TN, USA) and Gene Mapper software version 4.0 (Applied Biosystems). Band intensities were estimated using BioNumerics version 7.6 (Applied Maths, Belgium), and repeat units were then obtained according to the published allele numbering system^22,23^. Clustering analysis was performed using the same software based on the categorical coefficient and unweighted pair group using arithmetic averages method. The resulting genotypes were compared using the web-based *Brucella* 2016 MLVA database (http://mlva.u-psud.fr/) and published papers^23^. Advanced clustering analysis was based on the MLVA for categorical data in the BioNumerics system.

### Genomic sequencing and evolutionary relationships of *B. melitensis* QY1

A dominant epidemic isolate, *B. melitensis* QY1, was used for whole-genome sequencing. Genomic DNA was extracted with the sodium-dodecyl sulphate (SDS) method. The harvested DNA was detected by agarose gel electrophoresis and quantified by Qubit (Life Qubit ® Fluorescence Quantitative Instrument, USA). The genome of *B. melitensis* QY1 was sequenced by single-molecule real-time (SMRT) technology. Sequencing was performed at Beijing Novogene Bioinformatics Technology Co., Ltd. The low-quality reads were filtered by SMRT 2.3.0^25,26^, and the filtered reads were assembled to generate one contig without gaps (accession nos. CP022204.1-CP022205.1)^27^. The evolutionary relationships were evaluated in comparison with *B. melitensis* M28*, B. melitensis* M5-90*, B. melitensis* NI*, B. melitensis* bv. 1 str. 16M*, B. melitensis* bv. 3 str. Ether, *B. melitensis* biovar abortus 2308 and *B. melitensis* ATCC 23457^28^ (Table S3). Based on the whole genomes of nine selected *B. melitensis* strains, the amino-acid sequences of core genes were analyzed by the CD-HIT software for rapid clustering of similar proteins with a threshold of 50% pairwise identity and 0.7 length difference cutoff. A phylogenetic tree was constructed from the orthologous gene sequences by TreeBeST using the neighbor-joining method, and the number of bootstrap samples was 1000^29,30^.

### Statistical analysis

The data were transferred to Microsoft Excel and evaluated using a two-tailed unpaired *t*-test to compare the two groups; *P*-values <0.05 were considered to be significantly different.

## Electronic supplementary material

supplement Table S1(DOC 399 kb)

supplement Table S2(DOC 53 kb)

supplement Table S3(DOC 30 kb)
