# Supplementary material for: Enzootic situation and molecular epidemiology of Brucella in livestock from 2011 to 2015 in Qingyang, China
Source: Emerg Microbes Infect. 2018 Apr 4;7:58. doi: 10.1038/s41426-018-0060-y (PMC5882930; doi:10.1038/s41426-018-0060-y)
Supplement: Supplementary file 1 — supplement Table S1(DOC 399 kb) [file 41426_2018_60_MOESM1_ESM.doc]

Supplement Table S1 MLVA-11 data, biovars, regions, and year of isolation for *B. melitensis* isolates in China

| Strain | bru06 | bru08 | bru11 | bru12 | bru42 | bru43 | bru45 | bru55 | bru18 | bru19 | bru21 | Species-Bovars | Origin | Host | Year |
| --- | --- | --- | --- | --- | --- | --- | --- | --- | --- | --- | --- | --- | --- | --- | --- |
| QY1 | 1 | 5 | 3 | 13 | 2 | 3 | 3 | 2 | 4 | 41 | 8 | *B.melitensis* biovar 3 | Huanxian,Gansu | Sheep | 2016 |
| QY2 | 1 | 5 | 3 | 13 | 2 | 3 | 3 | 2 | 4 | 41 | 8 | *B.melitensis* biovar 3 | Huanxian,Gansu | Sheep | 2016 |
| QY3 | 1 | 5 | 3 | 13 | 2 | 3 | 3 | 2 | 4 | 41 | 8 | *B.melitensis* biovar 3 | Huanxian,Gansu | Sheep | 2016 |
| M1 | 3 | 4 | 2 | 13 | 4 | 2 | 3 | 3 | 5 | 36 | 6 | *B.melitensis* biovar 1 | Zhengning,Gansu | Sheep | 2016 |
| M2 | 1 | 5 | 3 | 12 | 2 | 2 | 3 | 2 | 4 | 41 | 8 | *B.melitensis* biovar 3 | Heshui,Gansu | Sheep | 2016 |
| M3 | 1 | 5 | 3 | 13 | 2 | 2 | 3 | 2 | 4 | 41 | 8 | *B.melitensis* biovar 3 | Huachi,Gansu | Sheep | 2016 |
| M5 | 1 | 5 | 3 | 13 | 2 | 2 | 3 | 2 | 4 | 41 | 8 | *B.melitensis* biovar 3 | Huanxian,Gansu | Sheep | 2016 |
| T05 | 1 | 5 | 3 | 13 | 2 | 2 | 3 | 2 | 4 | 41 | 8 | *B.melitensis* biovar 3 | Zhenyuan,Gansu | Sheep | 2016 |
| T06 | 1 | 5 | 3 | 13 | 2 | 2 | 3 | 2 | 4 | 41 | 8 | *B.melitensis* biovar 3 | Zhenyuan,Gansu | Sheep | 2016 |
| T08 | 1 | 5 | 3 | 13 | 2 | 2 | 3 | 2 | 4 | 41 | *8* | *B.melitensis biovar 3* | Huanxian,Gansu | Sheep | 2016 |
| CN-4 | 1 | 5 | 4 | 13 | 2 | 2 | 3 | 2 | 4 | 21 | 8 | *B.melitensis* biovar 2 | Shandong | Sheep | 1974 |
| CN-10 | 1 | 5 | 3 | 13 | 2 | 1 | 3 | 2 | 4 | 40 | 8 | *B.melitensis* biovar 3 | Shandong | Sheep | 2001 |
| CN-11 | 1 | 5 | 3 | 13 | 2 | 1 | 3 | 2 | 4 | 40 | 8 | *B.melitensis* biovar 3 | Shandong | Sheep | 2001 |
| CN-17 | 1 | 5 | 3 | 13 | 2 | 3 | 3 | 2 | 4 | 40 | 8 | *B.melitensis* biovar 3 | Inner Mongolia | Sheep | 1996 |
| CN-18 | 1 | 5 | 3 | 13 | 2 | 3 | 3 | 2 | 4 | 40 | 8 | *B.melitensis* biovar 3 | Inner Mongolia | Sheep | 2006 |
| CN-21 | 1 | 5 | 3 | 13 | 2 | 3 | 3 | 2 | 4 | 40 | 8 | *B.melitensis* biovar 3 | Xinjiang | Sheep | 2011 |
| CN-22 | 1 | 5 | 3 | 13 | 2 | 3 | 3 | 2 | 4 | 40 | 8 | *B.melitensis* biovar 3 | Xinjiang | Sheep | 2011 |
| CN-71 | 1 | 5 | 3 | 13 | 2 | 2 | 3 | 2 | 4 | 21 | 8 | *B.melitensis* biovar 3 | Ningxia | Sheep | 1973 |
| CN-75 | 1 | 5 | 3 | 13 | 2 | 2 | 3 | 2 | 4 | 40 | 8 | *B.melitensis* biovar 2 | Hainan | Sheep | 1972 |
| CN-79 | 1 | 5 | 3 | 13 | 2 | 2 | 3 | 2 | 4 | 40 | 8 | *B.melitensis* biovar 2 | Inner Mongolia | Sheep | 1963 |
| CN-80 | 1 | 5 | 3 | 13 | 2 | 2 | 3 | 2 | 4 | 40 | 8 | *B.melitensis* biovar 2 | Inner Mongolia | Sheep | 1973 |
| CN-81 | 1 | 5 | 3 | 13 | 2 | 2 | 3 | 2 | 4 | 40 | 8 | *B.melitensis* biovar 2 | Inner Mongolia | Sheep | 1993 |
| CN-87 | 1 | 5 | 3 | 13 | 2 | 2 | 3 | 2 | 4 | 40 | 8 | *B.melitensis* biovar 2 | Xinjiang | Sheep | 1978 |
| CN-126 | 1 | 5 | 3 | 13 | 2 | 2 | 3 | 2 | 4 | 40 | 8 | *B.melitensis* biovar 3 | Hebei | Sheep | 2007 |
| CN-127 | 1 | 5 | 3 | 13 | 2 | 2 | 3 | 2 | 4 | 40 | 8 | *B.melitensis* biovar 3 | Hebei | Sheep | 2007 |
| CN-128 | 1 | 5 | 3 | 13 | 2 | 2 | 3 | 2 | 4 | 40 | 8 | *B.melitensis* biovar 3 | Heilongjiang | Sheep | 1962 |
| CN-129 | 1 | 5 | 3 | 13 | 2 | 2 | 3 | 2 | 4 | 40 | 8 | *B.melitensis* biovar 3 | Henan | Sheep | 2003 |
| CN-151 | 1 | 5 | 3 | 13 | 2 | 2 | 3 | 2 | 4 | 40 | 8 | *B.melitensis* biovar 3 | Inner Mongolia | Sheep | 1965 |
| CN-152 | 1 | 5 | 3 | 13 | 2 | 2 | 3 | 2 | 4 | 40 | 8 | *B.melitensis* biovar 3 | Inner Mongolia | Sheep | 1989 |
| CN-153 | 1 | 5 | 3 | 13 | 2 | 2 | 3 | 2 | 4 | 40 | 8 | *B.melitensis* biovar 3 | Inner Mongolia | Sheep | 1991 |
| CN-154 | 1 | 5 | 3 | 13 | 2 | 2 | 3 | 2 | 4 | 40 | 8 | *B.melitensis* biovar 3 | Inner Mongolia | Sheep | 1991 |
| CN-155 | 1 | 5 | 3 | 13 | 2 | 2 | 3 | 2 | 4 | 40 | 8 | *B.melitensis* biovar 3 | Inner Mongolia | Sheep | 1991 |
| CN-156 | 1 | 5 | 3 | 13 | 2 | 2 | 3 | 2 | 4 | 40 | 8 | *B.melitensis* biovar 3 | Inner Mongolia | Sheep | 1992 |
| CN-157 | 1 | 5 | 3 | 13 | 2 | 2 | 3 | 2 | 4 | 40 | 8 | *B.melitensis* biovar 3 | Inner Mongolia | Sheep | 1992 |
| CN-158 | 1 | 5 | 3 | 13 | 2 | 2 | 3 | 2 | 4 | 40 | 8 | *B.melitensis* biovar 3 | Inner Mongolia | Sheep | 1992 |
| CN-159 | 1 | 5 | 3 | 13 | 2 | 2 | 3 | 2 | 4 | 40 | 8 | *B.melitensis* biovar 3 | Inner Mongolia | Sheep | 1992 |
| CN-160 | 1 | 5 | 3 | 13 | 2 | 2 | 3 | 2 | 4 | 40 | 8 | *B.melitensis* biovar 3 | Inner Mongolia | Sheep | 1993 |
| CN-161 | 1 | 5 | 3 | 13 | 2 | 2 | 3 | 2 | 4 | 40 | 8 | *B.melitensis* biovar 3 | Inner Mongolia | Sheep | 1995 |
| CN-162 | 1 | 5 | 3 | 13 | 2 | 2 | 3 | 2 | 4 | 40 | 8 | *B.melitensis* biovar 3 | Inner Mongolia | Sheep | 1995 |
| CN-163 | 1 | 5 | 3 | 13 | 2 | 2 | 3 | 2 | 4 | 40 | 8 | *B.melitensis* biovar 3 | Inner Mongolia | Sheep | 1995 |
| CN-164 | 1 | 5 | 3 | 13 | 2 | 2 | 3 | 2 | 4 | 40 | 8 | *B.melitensis* biovar 3 | Inner Mongolia | Sheep | 1995 |
| CN-165 | 1 | 5 | 3 | 13 | 2 | 2 | 3 | 2 | 4 | 40 | 8 | *B.melitensis* biovar 3 | Inner Mongolia | Sheep | 1996 |
| CN-166 | 1 | 5 | 3 | 13 | 2 | 2 | 3 | 2 | 4 | 40 | 8 | *B.melitensis* biovar 3 | Inner Mongolia | Sheep | 2006 |
| CN-167 | 1 | 5 | 3 | 13 | 2 | 2 | 3 | 2 | 4 | 40 | 8 | *B.melitensis* biovar 3 | Inner Mongolia | Sheep | 2006 |
| CN-168 | 1 | 5 | 3 | 13 | 2 | 2 | 3 | 2 | 4 | 40 | 8 | *B.melitensis* biovar 3 | Inner Mongolia | Sheep | 2006 |
| CN-174 | 1 | 5 | 3 | 13 | 2 | 2 | 3 | 2 | 4 | 40 | 8 | *B.melitensis* biovar 3 | Qinghai | Sheep | 1980 |
| CN-175 | 1 | 5 | 3 | 13 | 2 | 2 | 3 | 2 | 4 | 40 | 8 | *B.melitensis* biovar 3 | Shandong | Sheep | 1991 |
| CN-176 | 1 | 5 | 3 | 13 | 2 | 2 | 3 | 2 | 4 | 40 | 8 | *B.melitensis* biovar 3 | Shandong | Sheep | 2001 |
| CN-177 | 1 | 5 | 3 | 13 | 2 | 2 | 3 | 2 | 4 | 40 | 8 | *B.melitensis* biovar 3 | Shandong | Sheep | 2001 |
| CN-186 | 1 | 5 | 3 | 13 | 2 | 2 | 3 | 2 | 4 | 40 | 8 | *B.melitensis* biovar 3 | Shanxi | Sheep | 1979 |
| CN-187 | 1 | 5 | 3 | 13 | 2 | 2 | 3 | 2 | 4 | 40 | 8 | *B.melitensis* biovar 3 | Shanxi | Sheep | 1979 |
| CN-188 | 1 | 5 | 3 | 13 | 2 | 2 | 3 | 2 | 4 | 40 | 8 | *B.melitensis* biovar 3 | Shanxi | Sheep | 1984 |
| CN-189 | 1 | 5 | 3 | 13 | 2 | 2 | 3 | 2 | 4 | 40 | 8 | *B.melitensis* biovar 3 | Sichuan | Sheep | 1963 |
| CN-193 | 1 | 5 | 3 | 13 | 2 | 2 | 3 | 2 | 4 | 40 | 8 | *B.melitensis* biovar 3 | Xinjiang | Goat | 2011 |
| CN-194 | 1 | 5 | 3 | 13 | 2 | 2 | 3 | 2 | 4 | 40 | 8 | *B.melitensis* biovar 3 | Xinjiang | Sheep | 2005 |
| CN-195 | 1 | 5 | 3 | 13 | 2 | 2 | 3 | 2 | 4 | 40 | 8 | *B.melitensis* biovar 3 | Xinjiang | Sheep | 2007 |
| CN-196 | 1 | 5 | 3 | 13 | 2 | 2 | 3 | 2 | 4 | 40 | 8 | *B.melitensis* biovar 3 | Xinjiang | Sheep | 2011 |
| CN-197 | 1 | 5 | 3 | 13 | 2 | 2 | 3 | 2 | 4 | 40 | 8 | *B.melitensis* biovar 3 | Xinjiang | Sheep | 2011 |
| CN-198 | 1 | 5 | 3 | 13 | 2 | 2 | 3 | 2 | 4 | 40 | 8 | *B.melitensis* biovar 3 | Xinjiang | Sheep | 2011 |
| CN-199 | 1 | 5 | 3 | 13 | 2 | 2 | 3 | 2 | 4 | 40 | 8 | *B.melitensis* biovar 3 | Xinjiang | Sheep | 2011 |
| CN-200 | 1 | 5 | 3 | 13 | 2 | 2 | 3 | 2 | 4 | 40 | 8 | *B.melitensis* biovar 3 | Xinjiang | Sheep | 2011 |
| CN-201 | 1 | 5 | 3 | 13 | 2 | 2 | 3 | 2 | 4 | 40 | 8 | *B.melitensis* biovar 3 | Xinjiang | Sheep | 2011 |
| CN-202 | 1 | 5 | 3 | 13 | 2 | 2 | 3 | 2 | 4 | 40 | 8 | *B.melitensis* biovar 3 | Xinjiang | Sheep | 2011 |
| CN-203 | 1 | 5 | 3 | 13 | 2 | 2 | 3 | 2 | 4 | 40 | 8 | *B.melitensis* biovar 3 | Xinjiang | Sheep | 2011 |
| CN-204 | 1 | 5 | 3 | 13 | 2 | 2 | 3 | 2 | 4 | 40 | 8 | *B.melitensis* biovar 3 | Xinjiang | Sheep | 2011 |
| CN-205 | 1 | 5 | 3 | 13 | 2 | 2 | 3 | 2 | 4 | 40 | 8 | *B.melitensis* biovar 3 | Xinjiang | Sheep | 2011 |
| CN-206 | 1 | 5 | 3 | 13 | 2 | 2 | 3 | 2 | 4 | 40 | 8 | *B.melitensis* biovar 3 | Zhejiang | Goat | 2011 |
| CN-207 | 1 | 5 | 3 | 13 | 2 | 2 | 3 | 2 | 4 | 40 | 8 | *B.melitensis* biovar 3 | Zhejiang | Goat | 2011 |
| CN-220 | 1 | 5 | 3 | 13 | 2 | 2 | 3 | 2 | 4 | 20 | 8 | *B.melitensis* biovar 2 | Inner Mongolia | Sheep | 1963 |
| CN-226 | 1 | 5 | 3 | 13 | 2 | 2 | 3 | 2 | 4 | 20 | 8 | *B.melitensis* biovar 2 | Xinjiang | Sheep | 1978 |
| CN-237 | 1 | 5 | 3 | 13 | 2 | 2 | 3 | 2 | 4 | 20 | 8 | *B.melitensis* biovar 3 | Heilongjiang | Sheep | 1962 |
| CN-252 | 1 | 5 | 3 | 13 | 2 | 2 | 3 | 2 | 4 | 20 | 8 | *B.melitensis* biovar 3 | Ningxia | Goat | 1973 |
| CN-253 | 1 | 5 | 3 | 13 | 2 | 2 | 3 | 2 | 4 | 20 | 8 | *B.melitensis* biovar 3 | Ningxia | Goat | 1979 |
| CN-302 | 1 | 5 | 3 | 13 | 2 | 2 | 3 | 2 | 4 | 20 | 8 | *B.melitensis* biovar 3 | Qinghai | Goat | 1980 |
| CN-309 | 1 | 5 | 3 | 13 | 2 | 2 | 3 | 2 | 4 | 20 | 8 | *B.melitensis* biovar 3 | Shanxi | Sheep | 1979 |
| CN-310 | 1 | 5 | 3 | 13 | 2 | 2 | 3 | 2 | 4 | 20 | 8 | *B.melitensis* biovar 3 | Sichuan | Sheep | 1963 |
| CN-312 | 1 | 5 | 3 | 13 | 2 | 2 | 3 | 2 | 4 | 42 | 8 | *B.melitensis* biovar 3 | Inner Mongolia | Sheep | 1988 |
| CN-314 | 1 | 5 | 3 | 13 | 2 | 2 | 3 | 2 | 4 | 46 | 8 | *B.melitensis* biovar 3 | Inner Mongolia | Sheep | 2006 |
| CN-315 | 1 | 5 | 3 | 13 | 2 | 2 | 3 | 2 | 4 | 46 | 8 | *B.melitensis* biovar 3 | Inner Mongolia | Sheep | 2006 |
| CN-316 | 1 | 5 | 3 | 13 | 2 | 2 | 3 | 2 | 4 | 46 | 8 | *B.melitensis* biovar 3 | Inner Mongolia | Sheep | 2006 |
| CN-321 | 1 | 5 | 3 | 13 | 2 | 2 | 3 | 2 | 4 | 44 | 8 | *B.melitensis* biovar 3 | Inner Mongolia | Sheep | 1989 |
| CN-322 | 1 | 5 | 3 | 13 | 2 | 2 | 3 | 2 | 4 | 44 | 8 | *B.melitensis* biovar 3 | Inner Mongolia | Sheep | 1991 |
| CN-323 | 1 | 5 | 3 | 13 | 2 | 2 | 3 | 2 | 4 | 44 | 8 | *B.melitensis* biovar 3 | Inner Mongolia | Sheep | 1992 |
| CN-343 | 1 | 5 | 1 | 2 | 2 | 2 | 3 | 2 | 4 | 20 | 8 | *B.melitensis* biovar 3 | Heilongjiang | Human | 2013 |
| CN-344 | 1 | 5 | 3 | 13 | 3 | 2 | 3 | 2 | 4 | 20 | 8 | *B.melitensis* biovar 3 | Chongqing | Sheep | 2013 |
| CN-345 | 1 | 5 | 3 | 13 | 3 | 2 | 3 | 2 | 4 | 20 | 8 | *B.melitensis* biovar 3 | Chongqing | Sheep | 2013 |
| CN-346 | 1 | 5 | 3 | 13 | 3 | 2 | 3 | 2 | 4 | 20 | 8 | *B.melitensis* biovar 3 | Chongqing | Sheep | 2013 |
| CN-368 | 1 | 4 | 3 | 13 | 2 | 2 | 3 | 2 | 6 | 42 | 8 | *B.melitensis* biovar 3 | Ningxia | Sheep | 1979 |
| CN-369 | 1 | 5 | 3 | 13 | 2 | 2 | 3 | 2 | 6 | 42 | 8 | *B.melitensis* biovar 3 | Inner Mongolia | Sheep | 1992 |
| CN-376 | 1 | 4 | 3 | 13 | 2 | 3 | 3 | 2 | 3 | 20 | 8 | *B.melitensis* biovar 3 | Ningxia | Goat | 1979 |
