# Supplementary material for: Enzootic situation and molecular epidemiology of Brucella in livestock from 2011 to 2015 in Qingyang, China
Source: Emerg Microbes Infect. 2018 Apr 4;7:58. doi: 10.1038/s41426-018-0060-y (PMC5882930; doi:10.1038/s41426-018-0060-y)
Supplement: Supplementary file 2 — supplement Table S2(DOC 53 kb) [file 41426_2018_60_MOESM2_ESM.doc]

Supplement Table S2 Sample size and positive result in each year

| County | 2011 | | | 2012 | | | 2013 | | | 2014 | | | 2015 | | |
| --- | --- | --- | --- | --- | --- | --- | --- | --- | --- | --- | --- | --- | --- | --- | --- |
|  | Size | P size | Rate (%) | Size | P size | Rate (%) | Size | P size | Rate (%) | Size | P size | Rate (%) | Size | P size | Rate (%) |
| Xifeng | 2012 | 0 | 0 | 3393 | 3 | 0.09 | 5127 | 8 | 0.16 | 6908 | 104 | 1.51 | 15030 | 183 | 1.22 |
| Zhengning | 2271 | 5 | 0.22 | 4649 | 13 | 0.28 | 6153 | 84 | 1.37 | 7076 | 19 | 0.27 | 10671 | 122 | 1.14 |
| Ningxian | 4771 | 11 | 0.23 | 8268 | 39 | 0.47 | 9481 | 6 | 0.06 | 17273 | 220 | 1.27 | 16429 | 272 | 1.66 |
| Heshui | 4914 | 41 | 0.83 | 8660 | 238 | 2.75 | 13486 | 117 | 0.87 | 14270 | 228 | 1.6 | 21459 | 682 | 3.18 |
| Zhenyuan | 3583 | 12 | 0.33 | 6240 | 12 | 0.19 | 7299 | 63 | 0.86 | 12470 | 140 | 1.12 | 14921 | 743 | 4.98 |
| Qingcheng | 5318 | 25 | 0.47 | 10288 | 97 | 0.94 | 5790 | 94 | 1.62 | 13112 | 322 | 2.46 | 13610 | 426 | 3.13 |
| Huachi | 5305 | 6 | 0.11 | 10991 | 22 | 0.2 | 11053 | 139 | 1.26 | 14449 | 708 | 4.9 | 33459 | 1014 | 3.03 |
| Huanxian | 3938 | 14 | 0.36 | 9200 | 130 | 1.41 | 10923 | 4 | 0.04 | 25568 | 451 | 1.76 | 48580 | 4837 | 9.96 |
| Total | 32112 | 114 | 0.04 | 61689 | 554 | 0.9 | 69312 | 515 | 0.74 | 111126 | 2192 | 1.97 | 174159 | 8279 | 4.75 |
