# Supplementary material for: Enzootic situation and molecular epidemiology of Brucella in livestock from 2011 to 2015 in Qingyang, China
Source: Emerg Microbes Infect. 2018 Apr 4;7:58. doi: 10.1038/s41426-018-0060-y (PMC5882930; doi:10.1038/s41426-018-0060-y)
Supplement: Supplementary file 3 — supplement Table S3(DOC 30 kb) [file 41426_2018_60_MOESM3_ESM.doc]

Supplement Table S3 The reference genome information of *Brucella* strains

| Strain | Accession no. | | Length(bp) | |
| --- | --- | --- | --- | --- |
|  | [Chr I](../../../../C:/Users/Administrator/AppData/Local/youdao/DictBeta/Application/7.2.0.0703/resultui/dict/%3Fkeyword=chromosome) | Chr II | [Chr I](../../../../C:/Users/Administrator/AppData/Local/youdao/DictBeta/Application/7.2.0.0703/resultui/dict/%3Fkeyword=chromosome) | Chr II |
| *B.melitensis* biovar Abortus 2308 | NC_007618.1 | NC_007624.1 | 2,121,359 | 1,156,948 |
| *B.melitensis* ATCC 23457 | CP001488.1 | CP001489.1 | 2,125,701 | 1,185,518 |
| *B.melitensis* M28 | CP002459.1 | CP002460.1 | 2,126,133 | 1,185,615 |
| *B.m*elitensis M-90 | CP001851.1 | CP001852.1 | 2,126,451 | 1,185,778 |
| *B.melitensis* NI | CP002931.1 | CP002932.1 | 2,117,717 | 1,176,758 |
| *B.melitensis* bv. 1 str. 16M | CP007763.1 | CP007762.1 | 2,116,984 | 1,177,791 |
| *B.melitensis* bv. 3 str. Ether | CP007760.1 | CP007761.1 | 2,122,766 | 1,187,961 |
| *B.melitensis* strain QY1 | CP022204.1 | CP022205.1 | 2,125,648 | 1,185,604 |
